# Supplementary material for: An intravenous pancreatic cancer therapeutic: Characterization of CRISPR/Cas9n-modified Clostridium novyi-Non Toxic
Source: PLoS One. 2023 Nov 14;18(11):e0289183. doi: 10.1371/journal.pone.0289183 (PMC10645340; doi:10.1371/journal.pone.0289183)
Supplement: S1 Table — (DOCX) [file pone.0289183.s001.docx]

**SUPPORTING INFORMATION**

**Table S1.**

| **Sequence Name** | **Sequence (5’-3’)** | |
| --- | --- | --- |
| **HDR Upstream Arm** | **FWD** | act tct cca ccg taa act tct aaa |
|  | **REV** | gca cct aag gaa gca gaa gaa |
| **HDR Downstream Arm** | **FWD** | tcc tga atta cg gat ggc ttt at |
|  | **REV** | gag gta caa ggt gtg gg tatt g |
| ***RGD*** | aga gga gat | |
| ***GGA* Spacer** | gga gga gct | |
