# Supplementary material for: An intravenous pancreatic cancer therapeutic: Characterization of CRISPR/Cas9n-modified Clostridium novyi-Non Toxic
Source: PLoS One. 2023 Nov 14;18(11):e0289183. doi: 10.1371/journal.pone.0289183 (PMC10645340; doi:10.1371/journal.pone.0289183)
Supplement: S2 Table — (summarized from previously published work ref 20). (DOCX) [file pone.0289183.s002.docx]

**SUPPORTING INFORMATION**

**Table S2.**

| **Sample** | **Resulting Amplicons** | **Bands Post-*Eco*RV Digest** | **Positive for Genomic Modification?** |
| --- | --- | --- | --- |
| **WT** | 1 | 1 | Negative |
| **A** | 1 | 2 | Positive |
| **B** | 1 | 2 | Positive |
| **C** | 1 | 2 | Positive |
| **D** | 1 | 2 | Positive |
| **E** | 1 | 2 | Positive |
| **(-)** | 0 | 0 | Negative |
