# Supplementary material for: An intravenous pancreatic cancer therapeutic: Characterization of CRISPR/Cas9n-modified Clostridium novyi-Non Toxic
Source: PLoS One. 2023 Nov 14;18(11):e0289183. doi: 10.1371/journal.pone.0289183 (PMC10645340; doi:10.1371/journal.pone.0289183)
Supplement: S3 Table — X-ray photoelectron spectroscopy was conducted to assess the elements present on a borosilicate sild cover after corona plasma treatment and subsequent coating with integrin. Adequate presence of integrin was determined when silicate was no longer detectable and nitrogen content had reached its maximum. (DOCX) [file pone.0289183.s003.docx]

**SUPPORTING INFORMATION**

**Table S3.**

| **Atomic %** | | | | | | |
| --- | --- | --- | --- | --- | --- | --- |
| **Element** | **No Treatment** | **Plasma Treatment** | **PBS** | **5ug/mL α_v_β_3_** | **10ug/mL α_v_β_3_** | **20ug/mL α_v_β_3_** |
| **O** | 57.96 | 64.05 | 55.4 | 34.76 | 44.78 | 38.33 |
| **C** | 9.81 | 3.54 | 12.68 | 25.37 | 25.34 | 8.09 |
| **Si** | 23.2 | 25.08 | 18.67 | 1.69 | 0 | 0 |
| **P** |  |  | 2.53 | 4.15 | 8.07 | 5.82 |
| **N** |  |  |  | 5.34 | 7.02 | 7.22 |
| **Cl** |  |  | 14.63 | 14.63 | 3.75 | 5.05 |
| **K** | 2.5 | 2.3 | 1.92 | 1.28 | 1.84 | 1.7 |
| **Na** | 3.95 | 3.25 | 8.14 | 12.78 | 9.2 | 33.79 |
