# Supplementary material for: An intravenous pancreatic cancer therapeutic: Characterization of CRISPR/Cas9n-modified Clostridium novyi-Non Toxic
Source: PLoS One. 2023 Nov 14;18(11):e0289183. doi: 10.1371/journal.pone.0289183 (PMC10645340; doi:10.1371/journal.pone.0289183)
Supplement: S4 Table — (DOCX) [file pone.0289183.s004.docx]

**SUPPORTING INFORMATION**

**Table S4.**

| **Channel** | **Excitation/**  **Emission Wavelength** | **Name** | **Molecule Detected** | **Primary**  **Antibody Detection** | **Species** | **Manufacturer** | **Catalog Number** |
| --- | --- | --- | --- | --- | --- | --- | --- |
| DAPI | 359/461 | DAPI | DNA | N/A | N/A | Thermofisher | P36930 |
| Cy3 | 492/508 | MUC1 | Mucin 1 | Conjugated in house via kit | Armenian Hamster | Thermofisher | MA5-11202 |
| Cy5 | 651/670 | MPO | Neutrophils | Manufacturer Conjugated | Rabbit | Abcam | ab252131 |
| Cy7 | 756/779 | F4/80 | Murine Macrophages | Conjugated in house via kit | Rat | Novus | NB600-404 |
