# Supplementary material for: An intravenous pancreatic cancer therapeutic: Characterization of CRISPR/Cas9n-modified Clostridium novyi-Non Toxic
Source: PLoS One. 2023 Nov 14;18(11):e0289183. doi: 10.1371/journal.pone.0289183 (PMC10645340; doi:10.1371/journal.pone.0289183)
Supplement: S5 Table — (DOCX) [file pone.0289183.s005.docx]

**SUPPORTING INFORMATION**

**Table S5.**

| Sample | Tumor Implant | Tail Vein Injection | Tissue |
| --- | --- | --- | --- |
| 2 | KPC | WT | Pancreas |
| 3 | KPC | WT | Spleen |
| 26 | KPC | RGD-Mod | Spleen |
| 27 | KPC | RGD-Mod | Pancreas |
| 28 | KPC | RGD-Mod | Pancreatic Tumor |
| 31 | KPC | RGD-Mod | Lung |
| 32 | KPC | RGD-Mod | Heart |
| 37 | KPC | PBS | Liver |
| *E. coli* | n.a. | n.a. | n.a. |
| 39 | KPC | PBS | Lung |
| 42 | KPC | RGD-Mod | Spleen |
| 43 | KPC | RGD-Mod | Pancreas |
| 44 | KPC | RGD-Mod | Pancreatic Tumor |
| 45 | KPC | RGD-Mod | Liver |
| 47 | KPC | RGD-Mod | Lung |
| 50 | KPC | RGD-Mod | Spleen |
| 52 | KPC | RGD-Mod | Pancreatic Tumor |
| *E. coli* | n.a. | n.a. | n.a. |
| 59 | KPC | WT | Pancreas |
| 60 | KPC | WT | Pancreatic Tumor |
| 63 | KPC | WT | Lung |
| 66 | KPC | RGD-Mod | Pancreas |
| 69 | KPC | RGD-Mod | Lung |
| 70 | KPC | RGD-Mod | Heart |
| 71 | KPC | RGD-Mod | Brain |
| 74 | PBS | RGD-Mod | Kidney |
| *E. coli* | n.a. | n.a. | n.a. |
| 75 | PBS | RGD-Mod | Spleen |
| 76 | PBS | RGD-Mod | Pancreas |
| 77 | PBS | RGD-Mod | Liver |
| 78 | PBS | RGD-Mod | Kidney |
| 80 | PBS | RGD-Mod | Heart |
| 81 | PBS | RGD-Mod | Brain |
| 82 | PBS | RGD-Mod | Spleen |
| 83 | PBS | RGD-Mod | Pancreas |
| *E. coli* | n.a. | n.a. | n.a. |
| 85 | PBS | RGD-Mod | Kidney |
| 86 | PBS | RGD-Mod | Lung |
| 87 | PBS | RGD-Mod | Heart |
| 88 | PBS | RGD-Mod | Brain |
| 89 | KPC | WT | Spleen |
| 90 | KPC | WT | Pancreas |
| 96 | PBS | PBS | Spleen |
| 103 | PBS | RGD-Mod | Spleen |
| *E. coli* | n.a. | n.a. | n.a. |

**Table S5 (continued).** Key to the samples run in Figure S12.

| Sample | Tumor Implant | Tail Vein Injection | Tissue |
| --- | --- | --- | --- |
| 104 | PBS | RGD-Mod | Pancreas |
| 107 | PBS | RGD-Mod | Lung |
| 108 | PBS | RGD-Mod | Heart |
| 117 | PBS | WT | Spleen |
| 118 | PBS | WT | Pancreas |
| 121 | PBS | WT | Lung |
| 128 | PBS | PBS | Lung |
| *E. coli* | n.a. | n.a. | n.a. |
| 132 | PBS | RGD-Mod | Liver |
| 135 | PBS | RGD-Mod | Kidney |
| 140 | PBS | RGD-Mod | Blood |
| 141 | PBS | RGD-Mod | Urine |
| *E. coli* | n.a. | n.a. | n.a. |
