# Supplementary figures and images for: An intravenous pancreatic cancer therapeutic: Characterization of CRISPR/Cas9n-modified Clostridium novyi-Non Toxic
Source: PLoS One. 2023 Nov 14;18(11):e0289183. doi: 10.1371/journal.pone.0289183 (PMC10645340; doi:10.1371/journal.pone.0289183)

**SUPPORTING INFORMATION**

**
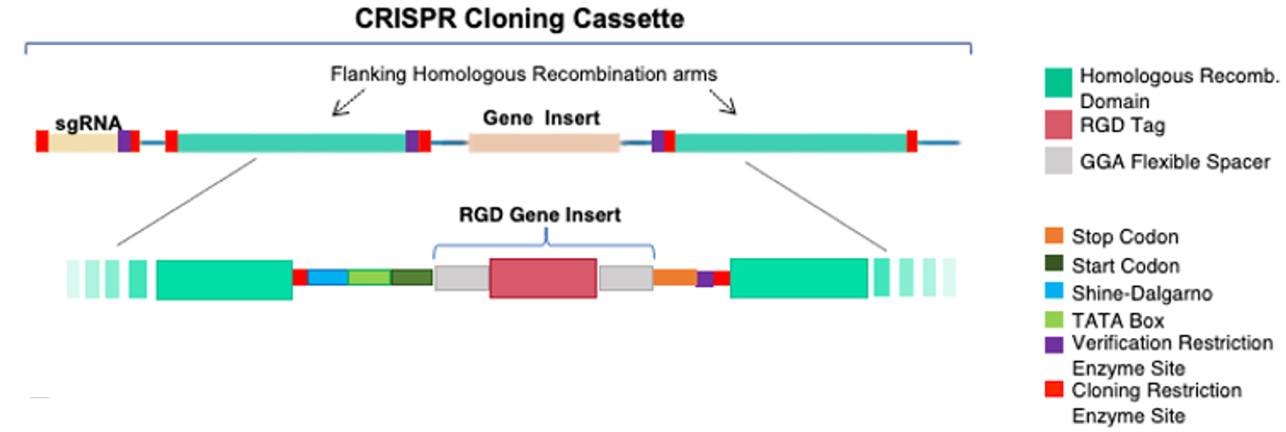
**

**Figure S1**

Supplement: S1 Fig — Schematic representation of the CRISPR cloning cassette utilized in pKMD002 for gene insertion. (DOCX) [file pone.0289183.s006.docx]

**SUPPORTING INFORMATION**


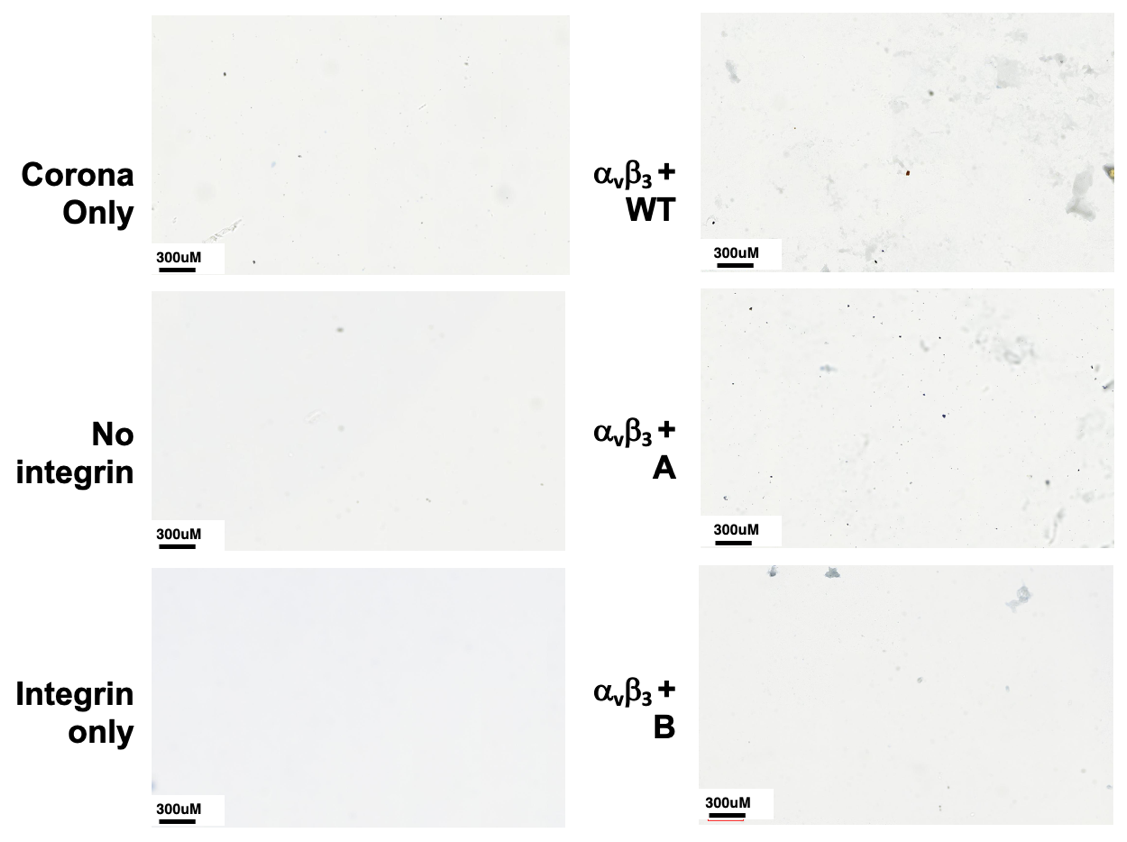


**Figure S2**.

Supplement: S2 Fig — (DOCX) [file pone.0289183.s007.docx]

**SUPPORTING INFORMATION**


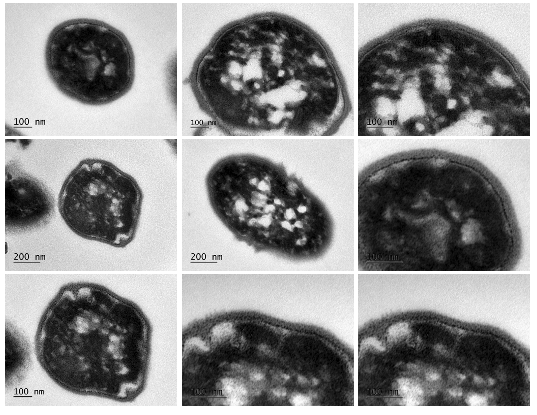


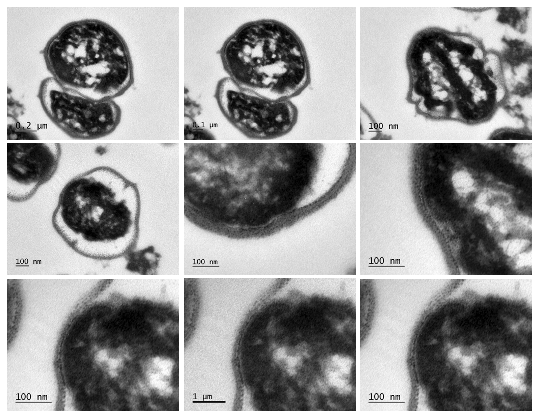


**Figure S3**.

Supplement: S3 Fig — (DOCX) [file pone.0289183.s008.docx]

**SUPPORTING INFORMATION**


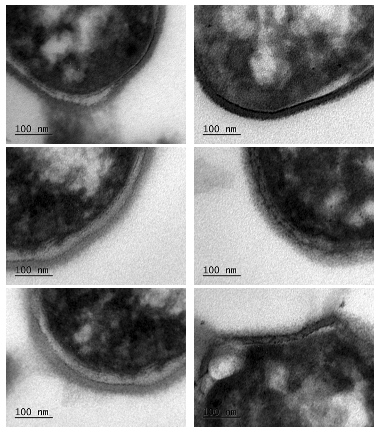
**
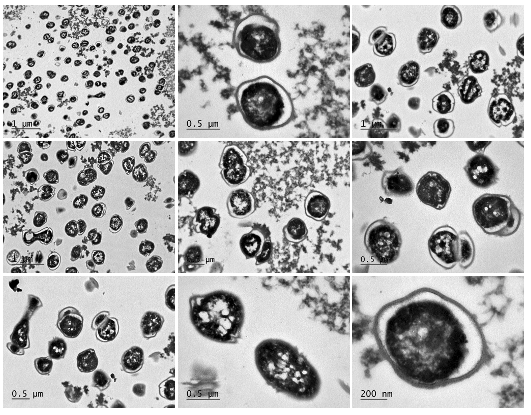
**
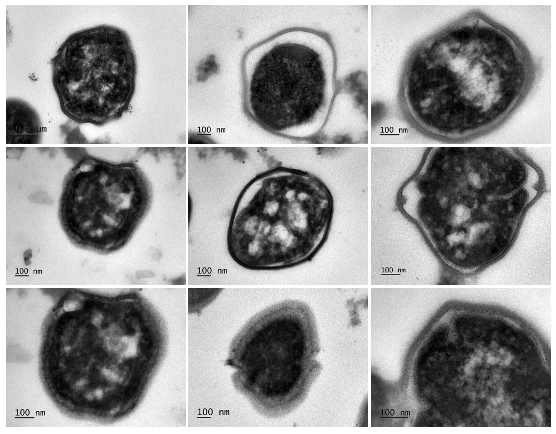


**Figure S4**.

Supplement: S4 Fig — (DOCX) [file pone.0289183.s009.docx]

**SUPPORTING INFORMATION**


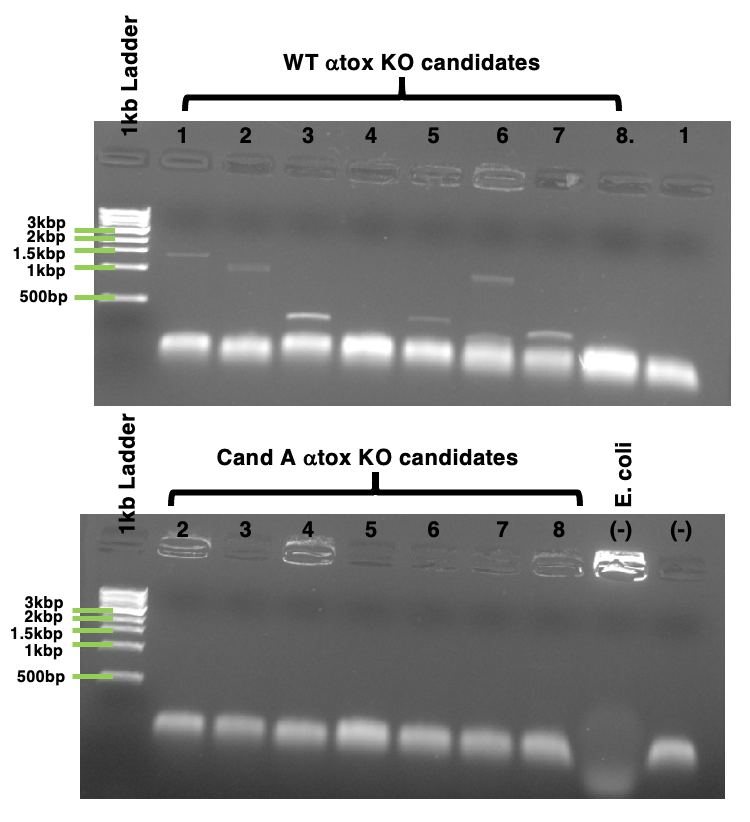


**Figure S5**.

Supplement: S5 Fig — In order to accomplish in vivo introduction without substantial toxicity, the α-toxin encoded phage DNA had to be knocked out in C. novyi that had already undergone successful genetic modification with RGD-encoding DNA. Upon knockout, PCR was conducted with primers specific to the α-toxin so that a lack of a band around 500bp represents a-toxin removal. (DOCX) [file pone.0289183.s010.docx]

**SUPPORTING INFORMATION**


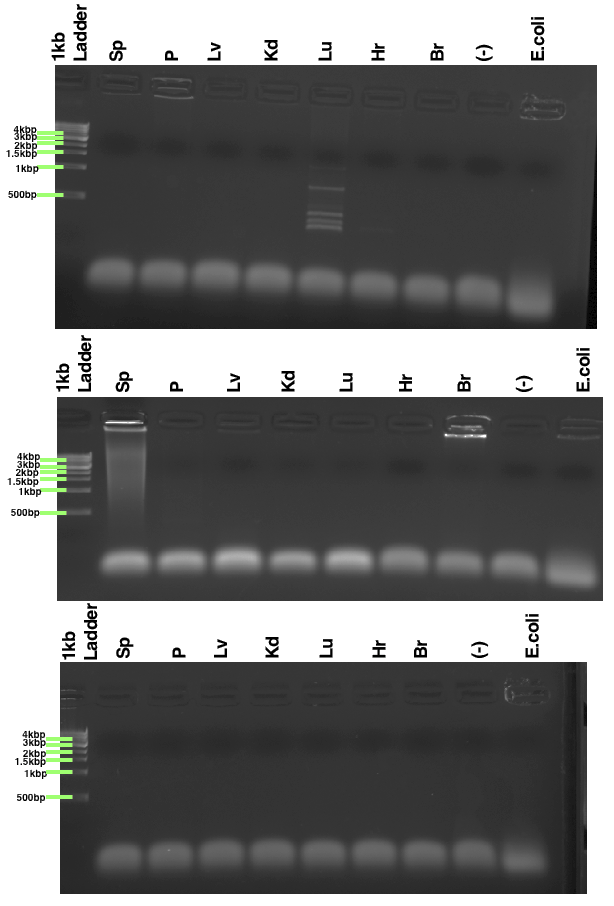


**Figure S6**.

Supplement: S6 Fig — (Sp–spleen, P–pancreas, Lv- liver, Kd- kidney, Lu- lung, Ht- heart, Br- brain, (-) no template control, E. coli DNA control). (DOCX) [file pone.0289183.s011.docx]

**SUPPORTING INFORMATION**


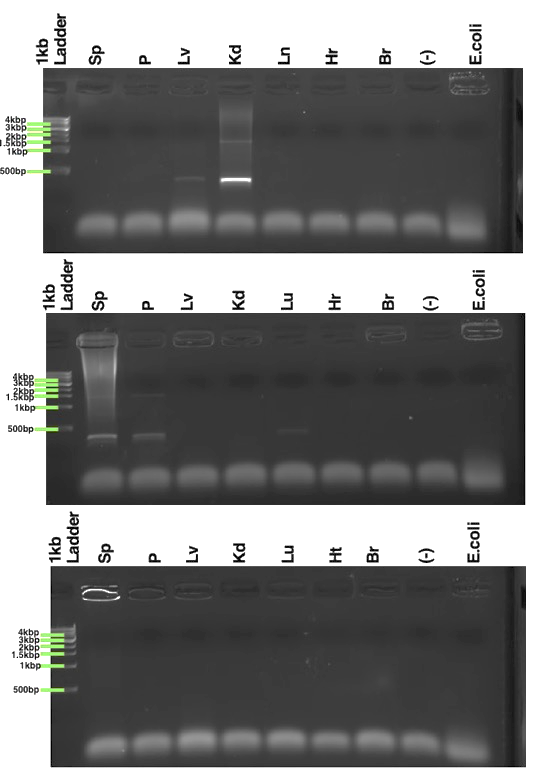


**Figure S7**.

Supplement: S7 Fig — (Sp–spleen, P–pancreas, Lv- liver, Kd- kidney, Lu- lung, Ht- heart, Br- brain, (-) no template control, E. coli DNA control). (DOCX) [file pone.0289183.s012.docx]

**SUPPORTING INFORMATION**


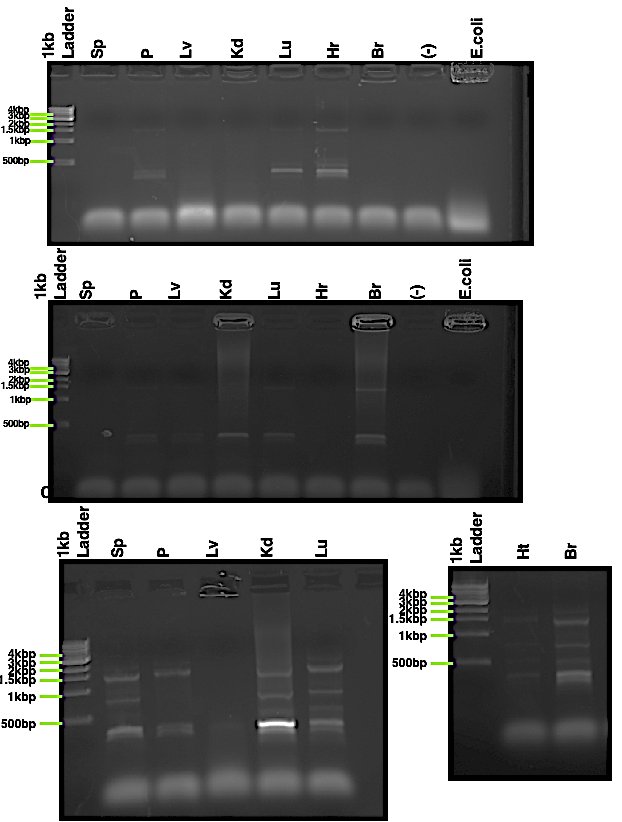


**Figure S8**.

Supplement: S8 Fig — (Sp–spleen, P–pancreas, Lv- liver, Kd- kidney, Lu- lung, Ht- heart, Br- brain, (-) no template control, E. coli DNA control). (DOCX) [file pone.0289183.s013.docx]

**SUPPORTING INFORMATION**


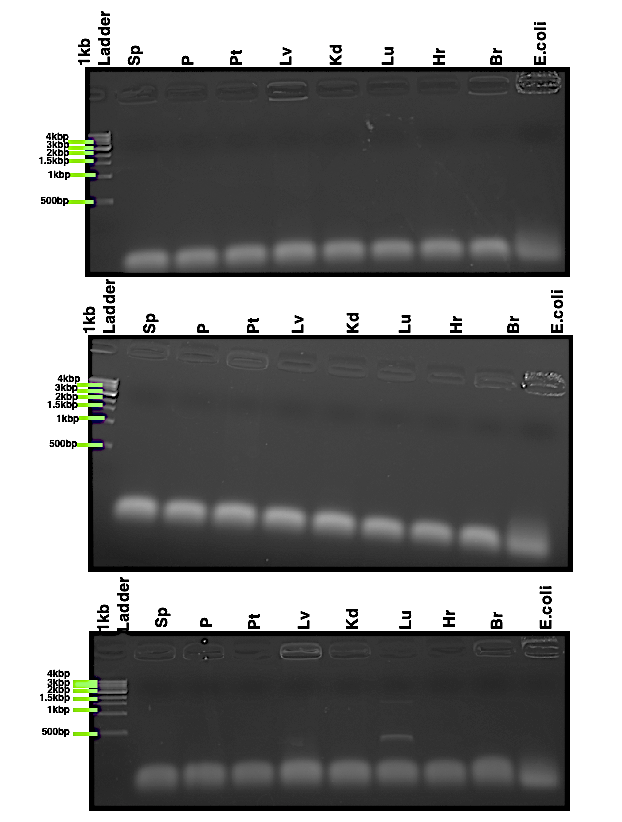


**Figure S9**.

Supplement: S9 Fig — (Sp–spleen, P–pancreas, Pt–pancreatic tumor, Lv- liver, Kd- kidney, Lu- lung, Ht- heart, Br- brain, (-) no template control, E. coli DNA control). (DOCX) [file pone.0289183.s014.docx]

**SUPPORTING INFORMATION**


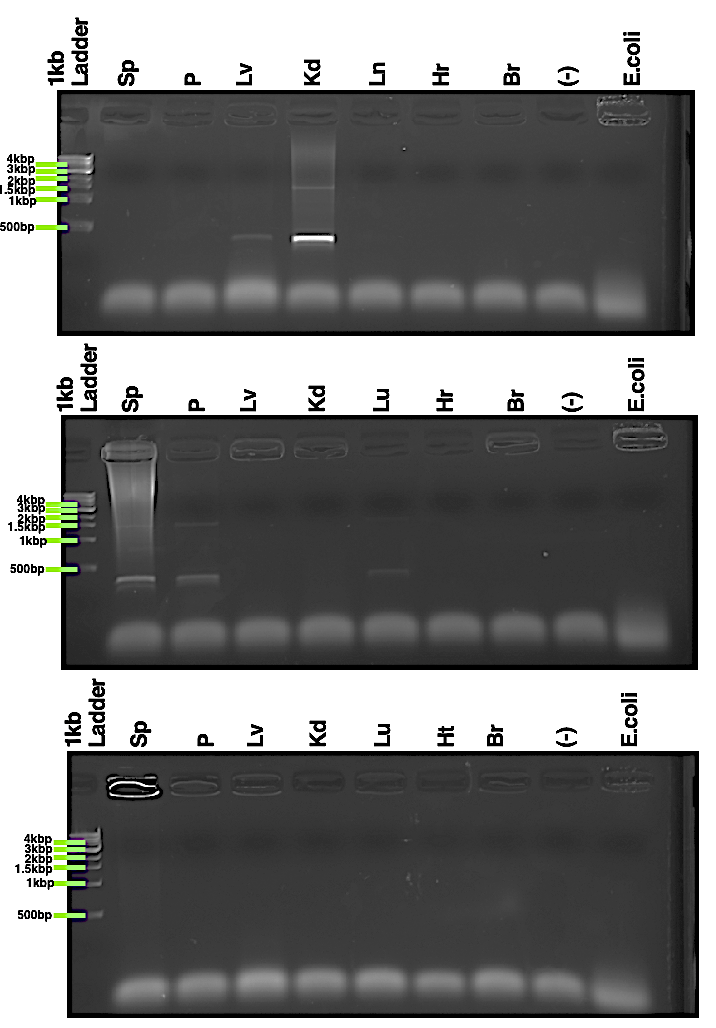


**Figure S10.**

Supplement: S10 Fig — (Sp–spleen, P–pancreas, Pt–pancreatic tumor, Lv- liver, Kd- kidney, Lu- lung, Ht- heart, Br- brain, (-) no template control, E. coli DNA control). (DOCX) [file pone.0289183.s015.docx]

**SUPPORTING INFORMATION**


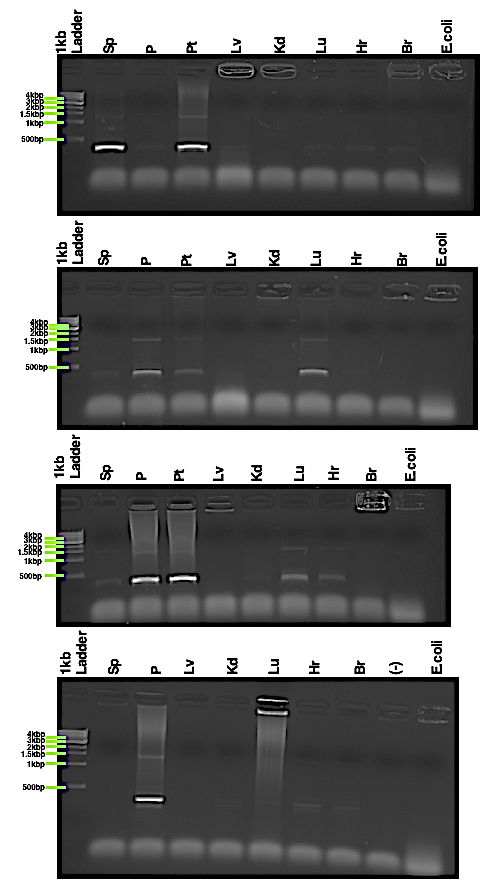


**Figure S11**.

Supplement: S11 Fig — (Sp–spleen, P–pancreas, Pt–pancreatic tumor, Lv- liver, Kd- kidney, Lu- lung, Ht- heart, Br- brain, (-) no template control, E. coli DNA control). (DOCX) [file pone.0289183.s016.docx]

**SUPPORTING INFORMATION**


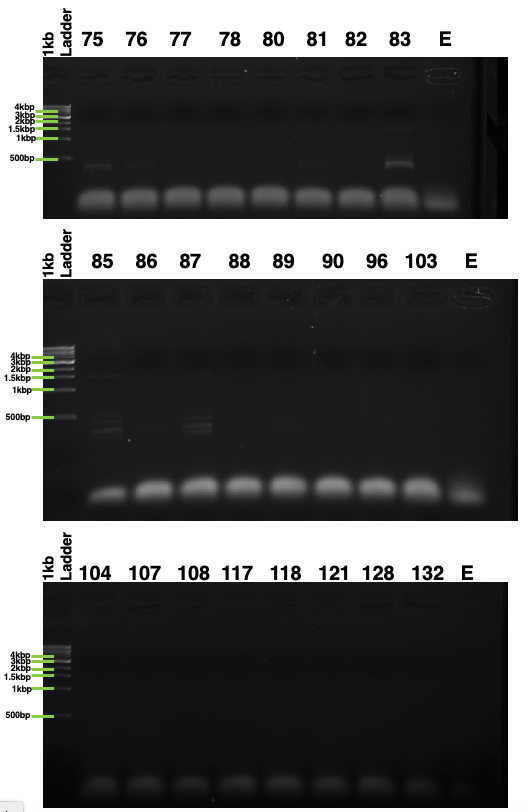

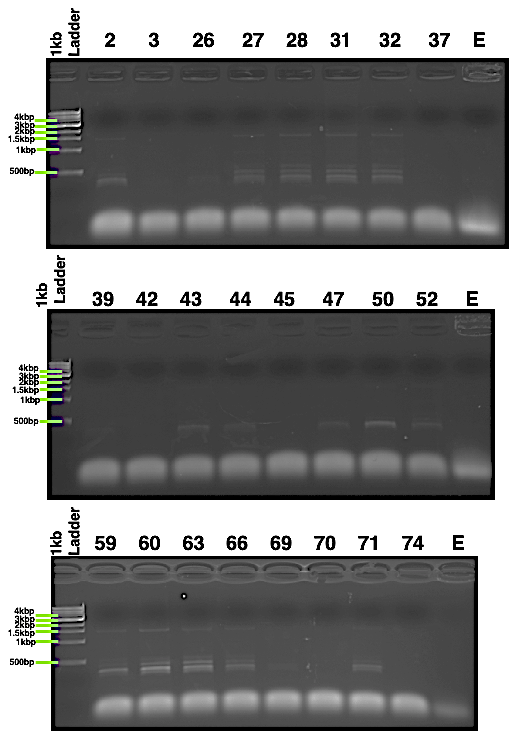


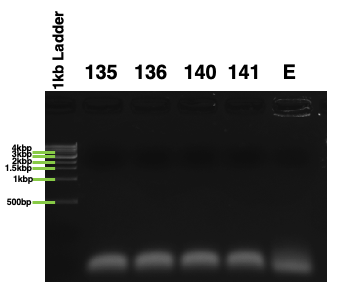


**Figure S12.**

Supplement: S12 Fig — (DOCX) [file pone.0289183.s017.docx]
